# Neoadjuvant Chemotherapy with Taxane and Platinum Followed by Radical Hysterectomy for Stage IB2-IIB Cervical Cancer: Impact of Histology Type on Survival

**DOI:** 10.3390/jcm8020156

**Published:** 2019-01-30

**Authors:** Koji Matsuo, Muneaki Shimada, Satoshi Yamaguchi, Junzo Kigawa, Hideki Tokunaga, Tsutomu Tabata, Junichi Kodama, Kei Kawana, Mikio Mikami, Toru Sugiyama

**Affiliations:** 1 Division of Gynecologic Oncology, Department of Obstetrics and Gynecology, University of Southern California, Los Angeles, CA 90089, USA; 2 Norris Comprehensive Cancer Center, University of Southern California, Los Angeles, CA 90033, USA; 3 Department of Obstetrics and Gynecology, Tottori University, Tottori 683-8504, Japan; kigawa@matsue-cityhospital.jp; 4 Department of Obstetrics and Gynecology, Tohoku University, Miyagi 980-8577, Japan; tokunagahideki@med.tohoku.ac.jp; 5 Department of Gynecologic Oncology, Hyogo Cancer Center, Hyogo 673-0021, Japan; s-yama@hp.pref.hyogo.jp; 6 Matsue City Hospital, Shimane 690-8509, Japan; 7 Department of Obstetrics and Gynecology, Mie University Hospital, Mie 514-8507, Japan; tabatat@clin.medic.mie-u.ac.jp; 8 Department of Obstetrics and Gynecology, Hiroshima City Hiroshima Citizens Hospital, Hiroshima 730-8518, Japan; kodama@cc.okayama-u.ac.jp; 9 Department of Obstetrics and Gynecology, Graduate School of Medicine, The University of Tokyo, Tokyo 113-8654, Japan; kkawana-tky@umin.org; 10 Department of Obstetrics and Gynecology, Faculty of Medicine, Nihon University School of Medicine, Tokyo 173-8610, Japan; 11 Department of Obstetrics and Gynecology, Tokai University, Kanagawa 259-1193, Japan; mmikami@is.icc.u-tokai.ac.jp; 12 Department of Obstetrics and Gynecology, Iwate Medical University, Iwate 020-0023, Japan; sugiyamatoru0802@yahoo.co.jp

**Keywords:** cervical cancer, neoadjuvant chemotherapy, radical hysterectomy, locally advanced, squamous, adenocarcinoma

## Abstract

The current study examined the histology-specific impact of neoadjuvant chemotherapy (NACT) with a taxane/platinum regimen on survival in women with locally-advanced cervical cancer who underwent radical hysterectomy. This nation-wide retrospective cohort study examined women with clinical stage IB2-IIB cervical cancer who received NACT prior to radical hysterectomy from 2004–2008 (*n* = 684). NACT type (taxane/platinum versus others) was correlated with survival based on histology: 511 squamous versus 173 non-squamous. Taxane/platinum chemotherapy use was more common in non-squamous compared to squamous tumors (53.8% versus 20.7%, *P* < 0.001). In both histology types, the taxane/platinum regimen was more frequently utilized over time (both, *P* < 0.01). Among squamous tumors, women who received taxane/platinum chemotherapy had survival comparable to those who received other regimens: 5-year rates for disease-free survival, 69.0% versus 70.1%, *P* = 0.98; and cause-specific survival, 80.0% versus 81.0%, *P* = 0.93. Similarly, in non-squamous tumors, disease-free survival (5-year rates: 60.4% versus 59.0%, *P* = 0.86) and cause-specific survival (74.7% versus 76.3%, *P* = 0.70) were similar. In conclusion, use of taxane/platinum regimens for NACT significantly increased during the study period. Irrespective of histology type, in women with clinical stage IB2-IIB cervical cancer who underwent NACT prior to radical hysterectomy, taxane/platinum regimens had a similar effect on survival compared to non-taxane/platinum regimens.

## 1. Introduction

Cervical cancer is the most common gynecologic malignancy in the world, and more than 527,000 women were estimated to have been diagnosed with this disease in 2012 [1]. The prognosis for women with cervical cancer largely relies on cancer stage and treatment modality. Generally, treatment type is based on disease stage, and women with locally-advanced disease (stage IB2-IIB) most commonly receive chemo-radiotherapy per the guideline recommendation in the United States [2]. In Japan, these women often undergo primary surgical treatment with radical hysterectomy [3,4].

Surgical treatment with radical hysterectomy for stage IB2-IIB cervical cancer imparts significantly increased perioperative morbidity compared to definitive radiotherapy-based treatment [5,6]. Moreover, postoperative radiotherapy, particularly with concurrent chemotherapy, following radical pelvic surgery even further compounds the risk of treatment-related toxicities [7]. For these reasons, the efficacy of NACT prior to radical hysterectomy has been examined to see if this reduces treatment-related toxicity and improves survival [8].

In a recent meta-analysis, NACT was reported to improve survival compared to surgical treatment alone [9]. Their study population represented mixed histology types, and histology-specific effects of NACT were not assessed [9]. Although non-squamous tumors, such as adenocarcinoma and adenosquamous tumors, remain relatively uncommon, they have proportionally increased among cervical cancers over time [10]. Moreover, these non-squamous tumors seem to have different tumor biology and possibly a different chemotherapy response compared to their squamous counterparts [10,11]. Therefore, histology-specific evaluation for NACT will be of use in the management of women with cervical cancer.

Mounting data have shown that taxane/platinum doublet chemotherapy regimens seem to be effective in cervical cancer [12,13,14,15]. Yet, histology-specific effectiveness of the taxane/platinum regimen as NACT has not been completely elucidated, and whether the response to this regimen in non-squamous tumors differs compared to squamous tumors remains understudied. The objective of the study was to examine the histology-specific association of NACT with a taxane/platinum doublet regimen and survival in women with stage IB2-IIB cervical cancer who subsequently underwent radical hysterectomy.

## 2. Materials and Methods

### 2.1. Data Source and Eligibility

This study is a secondary analysis of a prior surgical database that consisted of consecutive cases of women with clinical stage IB-IIB cervical cancer who had radical hysterectomy between 2004 and 2008 at 116 institutions [16,17,18,19,20]. Institutional Review Board approval was properly obtained for the study. Eligible criteria for the study were women with clinical stage IB2-IIB cervical cancer who underwent NACT followed by type III radical hysterectomy. Histology was limited to squamous, adenocarcinoma, or adenosquamous types. Exclusion criteria included clinical stage IB1 disease, no or unknown NACT status, or rare histology types.

### 2.2. Clinical Information

Among eligible cases, patient demographics (age, year of surgery, clinical tumor stage), surgical-pathological factors (histology type, parametrial involvement, nodal metastasis [pelvic and para-aortic], cervical stromal involvement, tumor size, lympho-vascular space invasion [LVSI], uterine corpus invasion, ovarian involvement, malignant peritoneal cytology), treatment type (institution type, NACT use [regimen and administered cycle], and adjuvant therapy type), and survival (disease-free survival [DFS], cause-specific survival [CSS], and anatomical recurrence site) were extracted. 

### 2.3. Study Definition

Cancer stage was based on the 2014 International Federation of Gynecology and Obstructs (FIGO) system [21]. Deep stromal invasion was defined as tumor invasion into the outer-half of the cervix per the Japanese Society of Gynecologic Oncology system [22]. Tumor size cutoff was based on the 2014 FIGO system [21]. Institution was grouped by the registered hospital volume for NACT as high (≥40 cases over 5 years), mid-high (20–39 cases), low-mid (10–19 cases), and low-volume (<10 cases), arbitrary grouped every 10–20 cases as adopting and modifying the results of a recent study (cutoff 20 cases) [23]. Adjuvant therapy was grouped as whole pelvic radiotherapy alone, concurrent chemo-radiotherapy, systemic chemotherapy alone, or both systemic chemotherapy and radiotherapy. 

DFS was defined as the time interval between NACT initiation and the first recurrence or death from cervical cancer. CSS was defined as the time interval between the NACT initiation and the death from cervical cancer. Cases with alive status at the last follow-up were censored for analysis. Anatomical site for the first recurrence was grouped as local or distant as previously described [16,17,18,19,20].

### 2.4. Statistical Consideration

The primary objective of the study was to examine the histology type-specific association of NACT regimen and survival in women with clinical stage IB2-IIB cervical cancer who received NACT prior to radical hysterectomy. The secondary objective of the study was to examine clinico-pathological factors related to NACT regimen choice. Two histology cohorts were examined for analysis: squamous type and non-squamous type (adenocarcinoma/adenosquamous). In each cohort, taxane/platinum regimens and other regimens were compared. 

Differences in continuous variables were assessed with the Student *t* test or Mann-Whitney *U* test as appropriate. Differences in categorical variables were assessed with the Fisher exact test or chi-square tests as appropriate. The Kaplan-Meier method was used to construct survival or cumulative incidence of recurrence curves, and the log-rank test was used to examine the differences among the curves. 

Cox proportional hazard regression models were used to adjust models to examine the association of NACT regimen and survival. Covariates with *P* < 0.05 on univariate analysis were included in the model to analyze the patient demographics that contributed to NACT regimen choice. Stepwise adjustment was performed to examine associations in each layer. Hazard ratios (HR) and 95% confidence intervals (CI) were used to express the magnitudes of statistical significance.

In a sensitivity analysis, the interactions of each NACT regimen type and survival were examined. Among those who received taxane/platinum regimen, cross-comparison of histology type was also assessed. A *P*-value of less than 0.05 was considered statistically significant (two-sided hypothesis). Statistical Package for Social Science software (IBM SPSS, version 24.0, Armonk, NY, USA) was used for all the analyses. 

## 3. Results 

### 3.1. Study Cohort

Among 6,003 cases in the surgical database, there were 2,445 cases that had radical hysterectomy for clinical stage IB2-IIB cervical cancer with known NACT status. Of those, 684 women received NACT prior to radical hysterectomy and represented the study cohort. 

### 3.2. Chemotherapy Choices per Histology Type

As the entire cohort, the most commonly used chemotherapy regimens were irinotecan-based (*n* = 223, 32.6%) followed by taxane/platinum (*n* = 199, 29.1%), mitomycin-based (*n* = 91, 13.3%), and fluorouracil-based (*n* = 71, 10.4%). Among the taxane/platinum regimen, carboplatin/paclitaxel (*n* = 92, 46.2%) was the most common combination followed by carboplatin/docetaxel (*n* = 60, 30.2%) and cisplatin/paclitaxel (*n* = 44, 22.1%). 

In squamous tumors, the most commonly used regimen was irinotecan-based (39.9%) followed by taxane/platinum (20.7%; Table 1). In non-squamous tumors, taxane/platinum (53.8%) was the most commonly used regimen followed by irinotecan-based and fluorouracil-based regimens (both, 11.0%). Across the two histology types, taxane/platinum doublet regimens were more commonly used in non-squamous tumors compared to squamous (53.8% versus 20.7%, *P* < 0.001). The median cycle number of administered NACT was 2, and more than 80% of cases received 1–2 cycles of NACT. Administered cycle number was similar between the two histology groups (*P* = 0.16). Among those who received postoperative chemotherapy, women who received the taxane/platinum regimen for NACT were more likely to again receive the taxane/platinum regimen (91.8% versus 28.8%, *P* < 0.001).

### 3.3. Patient Demographics per Histology Type

In both histology types, there was a significant increase in the utilization of taxane/platinum chemotherapy during the study period: in squamous type, 9.8% in 2004 to 32.2% in 2008 (2.3-fold relative increase, *P* = 0.005); and in non-squamous type, 45.8% in 2004 to 75.7% in 2008 (65.2% relative increase, *P* < 0.001; Table 2). Hospital volume was significantly associated with taxane/platinum use in both histology types, and in squamous tumors this regimen was more commonly used in low-mid and low volume centers, whereas in non-squamous tumors this regimen was more commonly used in low volume centers (both, *P* < 0.05). 

In squamous tumors, types of adjuvant therapy received were similar in those who received taxane/platinum chemotherapy regimens compared to other chemotherapy regimens. By contrast, in non-squamous tumors, women who received taxane/platinum NACT were less likely to receive adjuvant therapy in the first place (21.7% versus 78.3%), but if they did, were more likely to receive adjuvant therapy with concurrent chemo-radiotherapy (62.9% versus 37.1%) compared to those who received non-taxane/platinum NACT (*P* = 0.048).

### 3.4. Histology-specific Surgical-Pathological Factors

Results from hysterectomy specimens are shown in Table 3. In the squamous group, surgical-pathological factors were similar between those who had received taxane/platinum chemotherapy and those who had received other regimens (all, *P* > 0.05). In the non-squamous group, there was a higher frequency of large tumor size in the taxane/platinum group compared to the non-taxane/platinum group (57.3% versus 73.3%, *P* = 0.033) but other factors were similar between the regimens (all, *P* > 0.05).

### 3.5. Survival Outcome

The median follow-up time of censored cases was 5.7 years (interquartile range, 2.3) for the squamous cohort and 5.5 years (interquartile range, 2.3) for the non-squamous cohort. There were 158 and 73 survival events in the squamous and non-squamous groups, respectively, during the follow-up time. 

**Figure 1:**
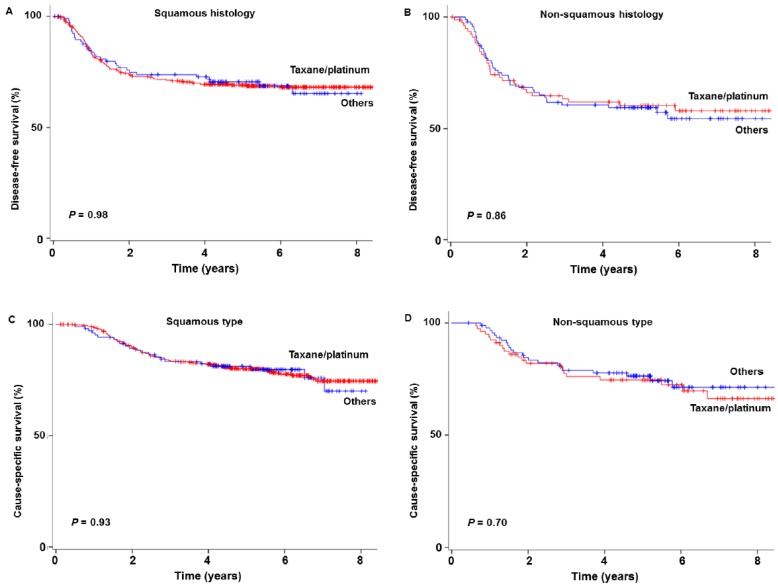
Survival outcomes based on histology types. Log-rank test for *P*-values. Survival curves are shown for disease-free (**A**) and cause-specific (**C**) in squamous type and disease-free (**B**) and cause-specific (**D**) in non-squamous type.

Among squamous tumors, women who received taxane/platinum chemotherapy had survival comparable to those who received other regimens (Figure 1): 5-year DFS rates, 69.0% versus 70.1% (*P* = 0.98); and 5-year CSS rates, 80.0% versus 81.0% (*P* = 0.93). After adjusting for year, surgical volume, and adjuvant therapy type (Table 4), the association between NACT type and survival remained insignificant among squamous tumors, and the taxane/platinum regimen was not associated with DFS (adjusted-HR 0.88, 95%CI 0.58–1.34) nor CSS (adjusted-HR 0.87, 95%CI 0.53–1.43) (both, *P* > 0.05). Among 109 women who did not receive any postoperative therapy, survival was similar between the taxane/platinum group and the non-taxane/platinum group (DFS *P* = 0.96, and CSS *P* = 0.25).

Similarly, among non-squamous tumors, DFS (60.4% versus 59.0%, *P* = 0.86) and CSS (74.7% versus 76.3%, *P* = 0.70) were similar between those who received taxane/platinum chemotherapy and those who received other regimens (Figure 1). The use of a taxane/platinum regimen was not associated with DFS (adjusted-HR 0.84, 95%CI 0.50–1.39) nor CSS (adjusted-HR 0.91, 95%CI 0.49–1.70) (both, *P* > 0.05). 

### 3.6. Sensitivity Analysis

A pairwise comparison was performed between the NACT regimens (Table 5). In squamous tumors, use of an NACT regimen was not associated with DFS (all, *P* > 0.05), including taxane/platinum versus irinotecan-based regimens (*P* = 0.65). Similarly, in non-squamous tumors, a NACT regimen was not associated with DFS (all, *P* > 0.05). Likewise, DFS was similar among three taxane/platinum regimens in the two histology groups (both, *P* > 0.05). Recurrence patterns for local and distant sites were similar between those who received taxane/platinum chemotherapy and those who received other regimens for both squamous and non-squamous tumors (all, *P* > 0.05; data not shown). 

Among women who received taxane/platinum NACT, those with non-squamous histology had poorer DFS compared to those with squamous histology although it did not reach statistical significance (HR 1.44, 95%CI 0.90–2.30, *P* = 0.12). Similarly, among women who received non-taxane/platinum regimen, those with non-squamous histology had poorer DFS compared to those with squamous histology although it did not reach statistical significance (HR 1.38, 95%CI 0.93–2.04, *P* = 0.11).

## 4. Discussion

Prior controlled trials that examined the effectiveness of NACT for locally-advanced cervical cancer were conducted with either squamous histology alone [24,25,26,27,28,29] or mixed histology types including both adenocarcinoma and adenosquamous histologies [8,30,31]. Some investigators have examined the effectiveness of NACT prior to radical hysterectomy in non-squamous tumors; however, this was in a non-randomized setting [32]. Collectively, trials examining the difference in NACT response by histology type are largely lacking in the literature.

Moreover, only one controlled trial previously examined the taxane/platinum doublet chemotherapy regimen as a NACT regimen, but the control group did not consist of those who received non-taxane/platinum regimens for NACT [26]. Outside of these trials, the effectiveness of a taxane/platinum doublet NACT regimen has been examined in cervical cancer; however, these prior studies had relatively small sample sizes (median size 35, range 14–115), lacked a control arm consisting of those who received non-taxane/platinum NACT regimens, or did not stratify treatment response by histology type [32,33,34,35,36,37,38,39,40]. Thus, while prior studies have suggested a possible utility of this regimen for NACT in cervical cancer, due to these limitations, interpretation of these results has been difficult and has been challenging to adopt in practice. 

As various studies have previously suggested that sensitivity to chemotherapy differs across histology subtypes in cervical cancer, and it is thought that non-squamous tumors have a more favorable chemotherapy response [41,42,43], examining the histology-specific response to NACT will be of value. Based on this rationale and background, our study is unique in demonstrating that taxane/platinum is comparable to non-taxane/platinum regimens for NACT in both squamous and non-squamous cervical cancer.

Taxane/platinum chemotherapy regimens have not been compared to other multi-agent regimens in the setting of NACT for locally-advanced cervical cancer. Outside of the NACT setting, this regimen was compared to various regimens for salvage chemotherapy [13,14,15]. In GOG-204, taxane/platinum regimens had superior survival compared to three other regimens, although it did not reach statistical significance [13]. In GOG-240, taxane/platinum regimens had overall survival similar to taxane/topotecan regimens regardless of bevacizumab use [15]. From this prospect, our study is similar to these studies. Of note, adenocarcinoma and adenosquamous cases made up 18–29% of their study populations, but a sub-analysis was not performed isolating non-squamous histology types. 

There has been a significant increase in the utilization of taxane/platinum regimens for NACT in cervical cancer, particularly in squamous tumors. The exact causality is not known, but it is speculated that mounting evidence supporting the effectiveness of this regimen as well as a favorable toxicity profile and treatment schedule may be partly responsible for the choice of this regimen by both providers and patients. This may be particularly applicable following a 2003 phase II trial the NACT regimen with paclitaxel/carboplatin seemed to be effective with acceptable toxicity [39]. As the vast majority of participants in this trial had squamous histology, it may be possible that increasing utilization of taxane/platinum regimen in squamous tumors was influenced by such a trial. 

Our study showed that nearly one third of patients developed disease recurrence in the first five years following the NACT-based approach. Indeed, our results externally validate a recent randomized trial that demonstrated similar survival statistics with the use of a taxane/platinum-based NACT regimens for stage IB2-IIB squamous cervical cancer (5-year DFS rates: 69.0% for ours and 69.3% for their trial, respectively) [26]. Notably, in this trial, the NACT-based approaches were inferior compared to definitive radiotherapy for early locally-advanced cervical cancer [26]. While the exact causality is unknown, it is speculated that the NACT-based approaches may provide inadequate treatment for local tissue [26]. 

Another biological plausible explanation for decreased survival with NACT use may be acquired chemotherapy-resistance. A recent meta-analysis demonstrated that reduction of high-risk tumor factors following NACT did not directly translate into an improvement in survival [44]. In ovarian cancer, use of NACT is suggested to increase platinum-resistance, possibly due to the presence of residual cancer stem cells after NACT [45,46]. In cervical cancer, a recent study reported that increased expression of a cancer stem cell marker after NACT was associated with decreased survival [47]. Overcoming chemoresistance in recurrent cervical cancer after NACT may be an emerging issue in the future [11]. 

A strength of our study is that our sample size is likely one of the largest reported in the literature. We performed a histology-specific analysis, which has considerable clinical implications in patient management. Due to the retrospective nature of this study, there may be missing confounding factors for analysis. For example, information regarding treatment decisions for NACT could not be assessed. Generally, women who undergo NACT may not be suitable for primary surgery due to decreased performance status or medical comorbidities. Similarly, the decision to choose the NACT regimen was not retrievable. Thus, while there seems to be inter-hospital variability in chemotherapy choice (Table 2), lack of this information makes this association unexplainable. While this study did not examine neoadjuvant radiotherapy use, this is a rare practice in Japan. Preoperative tumor factors were also not available for analysis, which have a definite impact on survival and constitute a critical limitation. 

A second limitation of this study is that this database lacks information regarding response rates for NACT and treatment-related complications and morbidity. Thus, a composite end-point for analysis combining survival and morbidity following NACT was not possible. A third limitation is that serum squamous cell carcinoma antigen levels were not available for analysis. This has been reported to be a predictor of NACT response in squamous cervical cancer [48]; however, it is unknown if these results may have been used in treatment decision-making regarding whether or not to proceed with NACT in these women. The fourth limitation of this study is that this database does not include information regarding tumor differentiation grade. A recent population-based study suggests that grade is an important prognostic factor in squamous cervical cancer, particularly in early-stage disease [49]. 

A fifth limitation is that while chemoresistance after NACT is an intriguing concept as previously described, our study does not have information regarding treatment type in recurrent cases of cervical cancer, so we are unable to examine this issue. The sixth limitation is that there were multiple NACT regimens administered in our study population. Because there is no current consensus regarding the optimal regimen for NACT, the large variation in chemotherapy regimens included in this study makes both interpretation and application difficult in practice. Some authors advocate the use of weekly paclitaxel, while others utilize a 3-week regimen for cervical cancer treatment. This analysis was not feasible in this study [50]. Finally, the lack of a control arm with primary surgery or radiotherapy limits the interpretation of our results to determine if NACT-based approaches have different survival effects compared to these other modalities as reported in a recent trial [26]. 

## 5. Conclusions

In conclusion, use of taxane/platinum regimens for NACT significantly increased during the study period. Irrespective of histology type, in women with clinical stage IB2-IIB cervical cancer who underwent NACT prior to radical hysterectomy, taxane/platinum regimens had a similar effect on survival compared to non-taxane/platinum regimens. The clinical utility of this study may be in the area of preoperative assessment and patient selection for NACT in women with stage IB2-IIB cervical cancer. Our study suggests that histology type is not a key factor for NACT response. Thus, it is reasonable to consider chemotherapy type based on the toxicity profile when NACT is considered for treatment. As taxane/platinum chemotherapy is a regimen that is relatively easy to administer and has a favorable toxicity profile compared to some other regimens, utility of this regimen merits further exploration.

## Figures and Tables

**Table 1:** Type and cycle of neoadjuvant chemotherapy.

Characteristic	All	Squamous	Non-Squamous	*P*-Value
Number	*n* = 684	*n* = 511	*n* = 173	
Regimen				**<0.001**
Irinotecan based	223 (32.6%)	204 (39.9%)	19 (11.0%)	
Taxane/platinum	199 (29.1%)	106 (20.7%)	93 (53.8%)	
Mitomycin based	91 (13.3%)	80 (15.7%)	11 (6.4%)	
Fluorouracil based	71 (10.4%)	52 (10.2%)	19 (11.0%)	
Platinum monotherapy	45 (6.6%)	36 (7.0%)	9 (5.2%)	
Ifosfamide based	26 (3.8%)	21 (4.1%)	5 (2.9%)	
Others	29 (4.2%)	12 (2.3%)	17 (9.8%)	
Cycles				0.16
1	195 (28.5%)	144 (28.2%)	51 (29.5%)	
2	382 (55.8%)	295 (57.7%)	87 (50.3%)	
3	84 (12.3%)	55 (10.8%)	29 (16.8%)	
≥4	23 (3.3%)	17 (3.3%)	6 (3.5%)	

Chi-square test for *P*-values. Significant *P*-values are emboldened.

**Table 2:** Pretreatment characteristics per histology type.

	Squamous			Non-Squamous		
Characteristic	Others	Taxane/Platinum	*P*-Value	Others	Taxane/Platinum	*P*-Value
Number	*n* = 405 (79.3%)	*n* = 106 (20.7%)		*n* = 80 (46.2%)	*n* = 93 (53.8%)	
Age	50.1 (SD ±11.3)	49.7 (SD ±12.7)	0.73	49.1 (SD ±11.6)	49.8 (SD ±10.6)	0.68
<50	191 (78.9%)	51 (21.1%)		42 (50.6%)	41 (49.4%)	
≥50	214 (79.6%)	55 (20.4%)		38 (42.2%)	52 (57.8%)	
Year			**0.005**			**<0.001**
2004	74 (90.2%)	8 (9.8%)		13 (54.2%)	11 (45.8%)	
2005	85 (80.2%)	21 (19.8%)		24 (68.6%)	11 (31.4%)	
2006	78 (75.7%)	25 (24.3%)		23 (59.0%)	16 (41.0%)	
2007	107 (82.3%)	23 (17.7%)		11 (28.9%)	27 (71.1%)	
2008	61 (67.8%)	29 (32.2%)		9 (24.3%)	28 (75.7%)	
Clinical stage			0.42			0.80
IB2	15 (68.2%)	7 (31.8%)		7 (38.9%)	11 (61.1%)	
IIA	74 (79.6%)	19 (20.4%)		15 (46.9%)	17 (53.1%)	
IIB	316 (79.8%)	80 (20.2%)		58 (47.2%)	65 (52.8%)	
Hospital volume			**0.001**			**0.032**
High	184 (78.0%)	52 (22.0%)		43 (50.0%)	43 (50.0%)	
Mid-high	137 (86.7%)	21 (13.3%)		30 (53.6%)	26 (46.4%)	
Low-mid	38 (62.3%)	23 (37.7%)		5 (25.0%)	15 (75.0%)	
Low	46 (82.1%)	10 (17.9%)		2 (18.2%)	9 (81.8%)	
Adjuvant therapy			0.08			**0.048**
None	87 (79.8%)	22 (20.2%)		5 (21.7%)	18 (78.3%)	
Radiotherapy alone	96 (82.1%)	21 (17.9%)		14 (50.0%)	14 (50.0%)	
CCRT	105 (84.0%)	20 (16.0%)		22 (62.9%)	13 (37.1%)	
Chemotherapy alone	82 (70.1%)	35 (29.9%)		35 (46.1%)	41 (53.9%)	
Both	10 (83.3%)	2 (16.7%)		3 (50.0%)	3 (50.0%)	

Mean (±SD) or number (percent per row) is shown. Student’s *t* test or chi-square test for *P*-values. Significant *P*-values are in bold. Abbreviations: CCRT, concurrent chemoradiotherapy; SD, standardized difference.

**Table 3:** Comparison of surgical-pathological factors per histology types.

	Squamous			Non-Squamous		
Characteristic	Others	Taxane/Platinum	*P*-Value	Others	Taxane/Platinum	*P*-Value
Number	*n* = 405	*n* = 106		*n* = 80	*n* = 93	
Parametria			0.13			0.15
Not involved	273 (67.4%)	80 (75.5%)		47 (58.8%)	65 (69.9%)	
Involved	132 (32.6%)	26 (24.5%)		33 (41.3%)	28 (30.1%)	
Tumor size			0.06			**0.033**
≤4 cm	155 (40.6%)	32 (30.2%)		32 (42.7%)	24 (26.7%)	
>4 cm	227 (59.4%)	74 (69.8%)		43 (57.3%)	66 (73.3%)	
Stromal invasion			0.99			0.50
Inner half	150 (37.8%)	40 (37.7%)		25 (33.8%)	26 (28.0%)	
Outer half	247 (62.2%)	66 (62.3%)		49 (66.2%)	67 (72.0%)	
LVSI			0.38			0.63
No	171 (43.2%)	51 (48.6%)		27 (35.5%)	37 (40.2%)	
Yes	225 (56.8%)	54 (51.4%)		49 (64.5%)	55 (59.8%)	
Uterine corpus			0.37			0.25
Not involved	334 (83.9%)	93 (87.7%)		50 (63.3%)	67 (72.0%)	
Involved	64 (16.1%)	13 (12.3%)		29 (36.7%)	26 (28.0%)	
Ovary			0.50			0.55
Not involved	394 (99.5%)	102 (99.0%)		76 (95.0%)	86 (92.5%)	
Involved	2 (0.5%)	1 (1.0%)		4 (5.0%)	7 (7.5%)	
Peritoneal cytology			0.99			0.73
No malignancy	156 (96.9%)	59 (98.3%)		39 (90.7%)	34 (87.2%)	
Malignancy	5 (3.1%)	1 (1.7%)		4 (9.3%)	5 (12.8%)	
Pelvic lymph node			0.42			0.19
Not involved	273 (68.8%)	79 (74.5%)		44 (56.4%)	64 (68.8%)	
Single metastasis	42 (10.6%)	11 (10.4%)		11 (14.1%)	7 (7.5%)	
Multiple metastases	82 (20.7%)	16 (15.1%)		23 (29.5%)	22 (23.7%)	
Para-aortic lymph node			0.78			0.69
Not involved	369 (95.8%)	103 (97.2%)		75 (97.4%)	88 (95.7%)	
Involved	16 (4.2%)	3 (2.8%)		2 (2.6%)	4 (4.3%)	

Number with percent per column is shown. Chi-square test or Mann-Whitney *U* test for *P*-values. Significant *P*-values are in bold. Abbreviations: LVSI, lympho-vascular space invasion.

**Table 4:** Adjustment models for neoadjuvant chemotherapy type and survival outcome.

Type	Adjustment Factors			Characteristics	Disease-Free Survival		Cause-Specific Survival	
	Year	Volume	Adjuvant		HR (95%CI)	*P*-Value	HR (95%CI)	*P*-Value
Squamous	+			Others	1		1	
				Taxane/platinum	1.01 (0.68–1.50)	0.95	1.00 (0.62–1.60)	0.99
	+	+		Others	1		1	
				Taxane/platinum	1.00 (0.67–1.49)	0.99	0.97 (0.60–1.57)	0.90
	+	+	+	Others	1		1	
				Taxane/platinum	0.88 (0.58–1.34)	0.56	0.87 (0.53–1.43)	0.58
Non-squamous	+			Others	1		1	
				Taxane/platinum	1.03 (0.63–1.69)	0.89	1.26 (0.68–2.32)	0.47
	+	+		Others	1		1	
				Taxane/platinum	1.03 (0.63–1.69)	0.91	1.19 (0.65–2.21)	0.57
	+	+	+	Others	1		1	
				Taxane/platinum	0.84 (0.50–1.39)	0.49	0.91 (0.49–1.70)	0.78

Cox proportional hazard regression models for adjustment models. Association of neoadjuvant chemotherapy type (taxane/platinum versus others) and survival was adjusted for the listed covariates in each layer: calendar year (continuous), surgical volume (low/low-mid, mid-high, and high), and adjuvant therapy types (none, radiotherapy alone, concurrent chemo-radiotherapy, systemic chemotherapy alone, and both). Abbreviations: HR, hazard radio; CI, confidence interval; year, calendar year; volume, surgical volume; and adjuvant, adjuvant therapy.

**Table 5:** Disease-free survival based on chemotherapy choice.

**Squamous**	**Non-Squamous**						
		**Taxane/Platinum**	**Irinotecan Based**	**Ifosfamide Based**	**Fluorouracil Based**	**Mitomycin Based**	**Platinum Alone**
	**Taxane/platinum**		0.70	0.78	0.48	0.22	0.22
	**Irinotecan based**	0.65		0.99	0.88	0.21	0.45
	**Ifosfamide based**	0.19	0.11		0.84	0.48	0.79
	**Fluorouracil based**	0.91	0.62	0.32		0.18	0.64
	**Mitomycin based**	0.94	0.59	0.25	0.98		0.11
	**Platinum alone**	0.44	0.28	0.62	0.60	0.57	

Log-rank test (pairwise) for *P*-values.
